# A Comparison of Aerobic Fitness Testing on a Swim Bench and Treadmill in a Recreational Surfing Cohort: A Pilot Study

**DOI:** 10.3390/sports6020054

**Published:** 2018-06-11

**Authors:** Hamzeh Khundaqji, Enad Samain, Mike Climstein, Ben Schram, Wayne Hing, James Furness

**Affiliations:** 1 Water Based Research Unit—Bond Institute of Health and Sport, Bond University, Gold Coast, QLD 4226, Australia; hamzeh.khundaqji@student.bond.edu.au (H.K.); enad.samain@student.bond.edu.au (E.S.); michael.climstein@scu.edu.au (M.C.); bschram@bond.edu.au (B.S.); whing@bond.edu.au (W.H.); 2 School of Health and Human Sciences, Southern Cross University, Lismore, NSW 2480, Australia; 3 Exercise, Health and Performance Faculty Research Group—Faculty of Health Sciences, The University of Sydney, Sydney, NSW 2006, Australia

**Keywords:** aerobic fitness, aerobic power, VO_2peak_, VO_2max_, ergometer, treadmill

## Abstract

The intermittent manner of surfing accentuates the importance of both the aerobic and anaerobic energy systems. Currently, the optimal method of assessing surfing-specific aerobic fitness is using a swim bench (SWB) ergometer; however, their limited availability presents a barrier to surfers wanting to know their maximal aerobic power (VO_2peak_). As a result, the aims of this pilot study were to determine the VO_2peak_ of recreational surfers using a new commercial SWB ergometer and to propose and examine the feasibility of a regression model to predict SWB ergometer VO_2peak_ values. A total of nine recreational surfers were assessed where body measurements were conducted followed by maximal aerobic capacity testing (swim bench and treadmill) to profile the cohort. Findings demonstrated that VO_2peak_ values were significantly greater (*p* < 0.001) on the treadmill compared to the SWB ergometer (*M* = 66.01 ± 8.23 vs. 37.41 ± 8.73 mL/kg/min). Peak heart rate was also significantly greater on the treadmill compared to the SWB ergometer. Multiple regression analysis was used to produce a model which predicted SWB VO_2peak_ values with an R^2^ value of 0.863 and an adjusted R^2^ value of 0.726. The physiological profiling of the recreational cohort coupled with a surfer’s predicted SWB VO_2peak_ value will allow for identification of surfing-specific aerobic fitness levels and evidence-based training recommendations.

## 1. Introduction

Surfing is regarded as part of a lifestyle and culture for those living on the coastal borders of countries like Australia and the United States. Today, surfing is enjoyed as an iconic pastime by 2.7 million Australians and 37 million individuals worldwide [1,2]. With its growth into a global multi-million-dollar industry and its recent induction into the Olympics, it is reasonable to assume that surfing’s impressive growth over the last decade will continue [3].

Using time-motion analysis, surfing has been subdivided into intermittent periods of arm paddling, prolonged periods of rest, and wave riding [3]. Monitoring of activity requirements for a 20 min heat in surfers using global positioning system (GPS) technology has revealed that paddling encompassed 42.6–44% of the total time with the majority of these paddling bouts (60%) ranging from 1 to 20 s long [4,5]. This intermittent manner of surfing accentuates the importance of both the aerobic and anaerobic energy systems [6]. The repeated high and low intensity paddling bouts within a heat may promote a high capacity of oxygen uptake, while allowing for adequate recovery between paddling spells [3]. Meir, et al. [5] reported that during one hour of recreational surfing, the mean heart rates during arm paddling represented 80% of the laboratory peak heart rate (HR_peak_) achieved by the surfers during a progressive swim bench (SWB) ergometer peak oxygen uptake (VO_2peak_) test. This suggests that a high level of aerobic fitness may be essential in surfing.

As evident through the existing body of research surrounding aerobic fitness testing in surfers, the preferred and ideal method of testing is completed with an SWB ergometer. Although dynamic leg exercise such as a cycle ergometer or treadmill are the most popular mode of exercise testing, research strongly supports the specificity of fitness testing [7]. For instance, VO_2peak_ values during arm work equate to approximately 70% of the values obtained during leg exercise [8]. However, athletes with a highly trained upper-body may achieve arm-crank values approaching 90% of their cycle VO_2peak_ [8].

Through a systematic review of the literature specific to aerobic fitness assessments in the surfing population, it is evident that the use of paddling ergometers to measure aerobic fitness in the elite cohort is steadily growing however remains limited in the recreational cohort [3,6,9,10] when compared to mainstream sports like kayaking [11,12,13,14,15,16,17] (see Appendix A for search strategy).

To date, only three studies have assessed recreational surfers using SWB ergometers [5,6,10]. Results from these studies ranged from 31.25 ± 6.31–54.20 ± 10.2 mL/kg/min indicating variations in VO_2peak_ across the recreational surfing cohort. Discrepancies in the results may be explained by differences in ergometers used and testing protocols employed. Previous studies measuring VO_2peak_ in surfing cohorts have employed a variety of equipment like the arm crank, tether board, treadmill and bicycle, which in theory could be the result of the limited availability of SWB ergometers [18,19,20].

**Figure 1:**
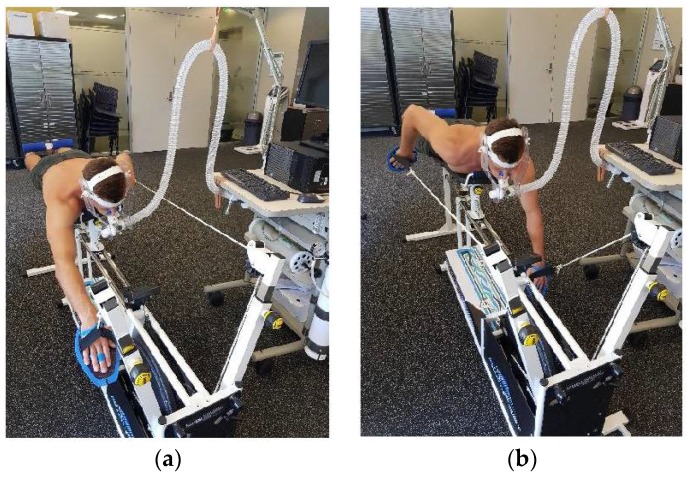
(**a**) Subject completing the catch phase of the paddling stroke on the swim bench ergometer setup in the laboratory; (**b**) Subject completing the pull phase of the paddling stroke on the swim bench ergometer setup in the laboratory.

Despite the treadmill and cycle ergometer being considered the optimal form of equipment for VO_2peak_ testing in both children and adults, the aforementioned differences between leg and arm work during maximal and submaximal exercise supports the use of a surfing-specific SWB ergometer [3,7]. However, the limited availability of SWB ergometers outside of leading sports performance institutes makes them difficult to access by recreational athletes. Given these concerns the aims of this pilot study were; to determine the VO_2peak_ of recreational surfers using a new commercial SWB ergometer (Figure 1) and to propose and examine the feasibility of a regression model to predict SWB ergometer VO_2peak_ values.

## 2. Materials and Methods

### 2.1. Subjects

This study involved nine male recreational surfers aged 18–42 years. To be classified as a recreational surfer, participants were to have a minimum of 12 months surfing experience, be currently surfing and list the sport as their main form of activity, and not be competing at higher than local club level. All testing of participants was conducted at the Bond University Institute of Health and Sport. Subjects were tested following their normal routine of sleep, nutrition and hydration levels. Ethics was granted through the Bond University Human Research Ethics Committee (RO1550) prior to commencement. Participants were informed of the associated risks and benefits of the study prior to signing an informed consent form.

### 2.2. Procedures

Testing was conducted by two experienced exercise scientists under the supervision of a senior researcher with expertise in maximal aerobic and anaerobic testing of recreational and professional surfers. Body measurements were conducted followed by the aerobic testing. All subjects underwent both a SWB ergometer and treadmill maximal oxygen consumption test. To minimize systematic bias and reduce the effects of fatigue the order of maximal oxygen consumption testing (i.e., treadmill or SWB) was randomized.

### 2.3. Body Measurements

Body measurements collected included height (EcoMed Seca, Hamburg, Germany), mass (Wedderburn, WM204, Sydney, Australia), with body mass index (BMI) subsequently calculated. Height was initially measured to the nearest 0.1 cm and body mass was measured to the closest 100 g with minimal clothing using a standard medical balance scale.

### 2.4. Aerobic VO_2peak_ Testing

Prior to commencing the incremental exercise testing, subjects were provided with standardized instructions on the use of the SWB ergometer and treadmill. Subsequently subjects completed a 2-min warm-up on the apparatus they were to be tested on that day. Warm-up on the SWB ergometer included paddling at below 10 watts, while warm-up on the treadmill included running at a speed of 8 or 10 km/h (subject preference) at an incline of 0 percent. This served to reduce the risk of injury and familiarize the subject with the equipment.

The subjects VO_2peak_ was obtained using incremental exercise testing on a SWB ergometer and treadmill. Peak oxygen consumption is considered the gold-standard for quantifying aerobic fitness [21]. SWB ergometry has previously been shown to be both valid and reliable to test peak aerobic levels in both recreational and competitive surfers [6,10]. Oxygen consumption was analyzed using an automated gas analysis system (Parvo Medics, TrueOne^®^, 2400, Sandy, UT, USA) (O_2_ analyzer, CO_2_ analyzer, pneumotach) that was calibrated with laboratory grade standard gasses (O_2_ and CO_2_) prior to each test. The SWB ergometer (KayakPro SwimFast, Miami, FL, USA) incremental test commenced at 10 watts, with increments of 10 watts every minute. This incremental test was adapted from a previously validated protocol by Furness, et al. [6]. Treadmill testing commenced at a speed of 8 or 10 km/h based upon the subject’s preference with an incline of 2% for the first minute [22]. At the start of the second minute the incline was increased to 4% then increased by 1% every subsequent minute [22]. The incremental treadmill test was developed in accordance with research by Sperlich, et al. [22] whose findings suggested that VO_2peak_ scores attained by employing individually designed treadmill exercise protocols allow the athlete to pace himself or herself according to their present biological state thus achieving higher VO_2peak_ values when compared with standardized protocols.

The testing termination criterion was based upon the ACSM guidelines for exercise testing and prescription [23]. Testing was terminated if age-predicted maximal heart rate was exceeded, respiratory exchange ratio (RER) reached greater than 1.5, oxygen consumption did not increase concurrently with power output, required power output was not maintained for greater than 10 s, volitional exhaustion was achieved, or any symptoms of chest pain were expressed by the participant. Heart rates (HR) were monitored throughout testing via telemetry using a Polar Team Pro HR sensor (Polar H7 Bluetooth HR Sensor) connected to Polar Team Pro software, which was interfaced with the Parvo Medics system. 

### 2.5. Statistical Analysis

Statistical analyses were performed using SPSS (Version 25.0; IBM Corp, Armonk, NY, USA). Normal distribution of the data was confirmed through a Shapiro-Wilks test and visual inspection of box plots, normal Q-Q plots and frequency histograms. Descriptive statistics including means (M), standard deviations (SD) and ranges were calculated for key performance variables (VO_2peak_, HR_peak_, percent of age predicted HR_max_ and peak aerobic power). A paired sample *t*-test was used to determine whether there was a statistical mean difference between participants VO_2peak_ scores on the SWB ergometer and treadmill. A multiple regression analysis was also conducted to produce a model to predict SWB ergometer VO_2peak_ scores. Prior to analyzing the data, the eight assumptions of multiple regression were considered and satisfied to ensure accuracy of the predictive model [24]. These assumptions are addressed in the results section. To assess the validity of the multiple regression model, a one-sample *t*-test was used to determine whether the mean difference between the two measures (SWB ergometer VO_2peak_ values versus predicted SWB ergometer VO_2peak_ values) were statistically different from zero. The level of agreement between the two measures was then represented through a Bland Altman plot with the associated 90% limits of agreement.

## 3. Results

### 3.1. Recreational Surfer Body Measurements and Aerobic Profile

All nine subjects successfully completed all body measurements and maximal oxygen consumption tests (treadmill and SWB) without incident. The body measurements and surfing experience of the surfers are listed in Table 1. Surfers had a mean BMI in the normal range (BMI 20.0 to ≤ 24.99 kg/m^2^) and a mean surfing experience of greater than 12 years. Subjects were surfing 5.22 h each week. Descriptive statistics of physical attributes and experience as well as key performance variables are presented in Table 1 and Table 2, respectively. Results of the paired samples *t*-tests in Table 2 demonstrate significant differences (*p* < 0.05) between the means of treadmill and SWB ergometer key performance variables.

The mean relative peak aerobic capacity for the recreational surfers on the treadmill was 66.01 mL/kg/min (range 49.0–70.1 mL/kg/min), which is significantly (*p* < 0.001) greater (+76.4%) than the SWB (M 37.41 mL/kg/min, range 24.0–43.6 mL/kg/min). Participants also had a significantly greater (+11.8%, *p* < 0.003) age-predicted HR_peak_ on the treadmill as compared to the SWB.

### 3.2. Development of a Multiple Regression Model

Prior to generating the multiple regression model, SPSS was used to analyze the data and ensure all appropriate multiple regression assumptions were satisfied. Linearity of variables was assessed and verified by partial regression plots and a plot of studentized residuals against the predicted values. Independence of residuals was also assessed and verified by a Durbin–Watson statistic of 2.466. Homoscedasticity was verified through the visual inspection of a plot of studentized residuals versus unstandardized predicted values. There was no evidence of multicollinearity, as assessed by a tolerance value greater than 0.1. When analyzing studentized deleted residuals values, leverage points and Cook’s distances, two participants were classified as outliers and were therefore excluded from the multiple-regression analysis as recommended by Laerd Statistics [24]. When analyzing the remaining participants (*n* = 7), there was no studentized deleted residuals greater than ±3 standard deviations, no leverage points greater than 0.2, and values for Cook’s distance above 1. The assumption of normality was met as previously discussed in the statistics section. The regression equation produced is presented below with a summary of the multiple regression analysis presented in Table 3. This multiple regression model predicted SWB ergometer VO_2peak_ scores with an R^2^ value of 0.863 and an adjusted R^2^ value of 0.726.

Swim Bench VO_2peak_ (mL/kg/min) = −33.027 +(0.480 × surf experience years) + (3.968 × hours surfed per week) + (0.699 × treadmill VO_2peak_).

### 3.3. Validity Assessment of SWB Ergometer Predictive Model: SWB versus Predicted SWB VO_2peak_ Values

**Figure 2:**
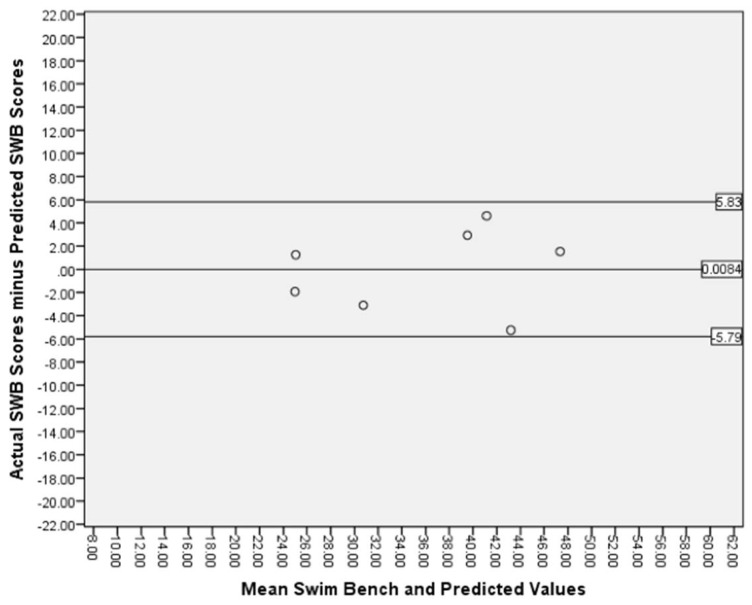
Bland Altman Plot displaying mean difference between the two measures and 90% CI.

A one-sample *t*-test determined the mean difference (0.0084 mL/kg/min) between the two measures (SWB ergometer VO_2peak_ values versus predicted SWB ergometer VO_2peak_ values) was not statistically (*p* > 0.05) different from zero. Bland–Altman plots with the associated 90% limits of agreement (5.83 and −5.79) are presented in Figure 2.

## 4. Discussion

The purpose of this study was to determine the VO_2peak_ of recreational surfers using a new commercial SWB ergometer and to propose a regression model to predict SWB ergometer VO_2peak_. Both aims have been satisfied and are discussed in detail below.

### 4.1. Body Measurements

This study has provided a profile of the recreational surfing cohort, which included body measurements of height, mass and key performance variables (HR_peak_, VO_2peak_, aerobic power output). Participants in this study had a mean age of 32.78 (±6.91) years versus 18 (±2.0) and 21.2 (±2.7) by Loveless and Minahan [10] and Meir, et al. [5] respectively. With respect to height of the male recreational surfing population, the mean height in the present study was 176.90 (±3.97) cm, which was slightly less than previous studies. Loveless and Minahan [10] investigated eight recreational surfers and reported a mean height of 179 (±11.0) cm. This is similar to the mean height of 180.13 (±7.54) cm reported by Furness, et al. [6] in 47 recreational surfers.

Mean body mass presented with similarities and discrepancies to previous research. In the present study, male recreational surfers had a mean body mass of 78.37 (±10.16) kg which was similar to the mean body mass of 77.42 (±10.69) kg reported by Furness, et al. [6] (77.42 ± 10.69). Previous research by Meir, et al. [5] and Loveless and Minahan [10] found mean mass to be slightly less with the authors reporting values of 68.9 (±5.67) kg and 66.8 (±13.00) kg respectively. The discrepancy in mean body mass reported in the present study and those by Meir, et al. [5] and Loveless and Minahan [10] could potentially be attributed to body composition changes that occur with aging such as increased fat mass [25].

### 4.2. Aerobic Profiles

Surfing is an activity characterized by intermittent exercise bouts of varying intensities and durations that requires exceptional aerobic fitness [3]. The use of time-motion analysis to monitor the activity requirements of a 20-min heat in surfers using GPS technology has revealed that paddling bouts account for 42.6–44% of the heat emphasizing the importance of the aerobic energy system in surfers [4,5].

The findings from this study established that recreational surfers had mean VO_2peak_ (±SD) values of 66.01 (±8.23) and 37.41 (±8.73) mL/kg/min on the treadmill and SWB respectively. When these values were compared to those of recreational runners and swimmers it was found that recreational surfers were capable of achieving larger VO_2peak_ values on the treadmill. Research by Gillen, et al. [26] assessed 11 recreational runners with a mean age of 34.1 years and found them to have a mean VO_2peak_ of 58.4 ± 7.8 mL/kg/min which is less than the mean value of this study’s recreational surfing cohort. Kimura, et al. [27] found recreational swimmers to have a mean VO_2peak_ of 58.3 ± 4.2 mL/kg/min on a treadmill, which was also less than the values achieved by the recreational surfing cohort in this study. It is theorized that surfing’s intermittent nature of high and low intensity bouts influence adaptions in physiological variables that impact aerobic endurance performance such as maximal oxygen uptake (VO_2peak_), lactate threshold, and work economy [28,29]. It has been demonstrated by previous research that high-aerobic intensity training otherwise known as high intensity interval training (HIIT) results in significantly increased absolute VO_2peak_ values when compared to alternative training methods such as long slow distance running and lactate running [29,30].

The SWB ergometer findings have similarities [10] and discrepancies [5,6] with previous research conducted on recreational surfing cohorts. The SWB ergometer VO_2peak_ values from this study are similar to those reported by Loveless and Minahan [10] (37.8 ± 4.5 mL/kg/min). These findings differ to those reported by Meir, et al. [5] (54.20 ± 10.2 mL/kg/min) and greater than those reported by Furness, et al. [6] (37.41 ± 8.73 mL/kg/min). The authors of this study hypothesize that the differences in VO_2peak_ could be attributed to the type of SWB ergometer used. The SwimFast ergometer used in this study differs from the Vasa and Repco ergometers used in previous studies as it allows for torsional roll (≈30°), which enables the recruitment of additional muscle groups in the thoracic region. This recruitment of additional muscle groups could lead to an increase in oxygen consumption resulting in higher VO_2peak_ values. The effects of the torsional roll on VO2_peak_ are further evident when examining the mean age of the cohorts used in the present study and previous research. The mean age for the present study was 32.78 (±6.91) yrs compared to younger subjects of 18.0 yrs (±2.0) and 26.50 yrs (±5.28) by Loveless and Minahan [10] and Furness, et al. [6], respectively. Aging is typically associated with a progressive decline in the capacity for physical activity due to the reduction in maximal rate of oxygen utilization [31]. However, as evident by the findings of this study, the increased age of our cohort did not result in reduced VO_2peak_ values when compared to the studies by Loveless and Furness. This further supports the theory that the torsional roll of the SwimFast ergometer allows for the use of larger muscle groups and consequently resulting in a greater need for oxygen.

To date, limited research has compared VO_2peak_ values obtained from SWB ergometer to those obtained from a treadmill [18]. When comparing VO_2peak_ from tethered board paddling, prone hand cranking and treadmill running, Lowdon, et al. [18] found that the peak oxygen consumption produced by running on a treadmill were the greatest. Previous research has demonstrated that aerobic capacity during arm work was considerably lower than utilizing the legs [8,32,33]. Stenberg, et al. [33] compared arm work to leg work and found that VO_2peak_ during arm ergometry was only 66% of that achieved by sitting leg cycle ergometry. This relates to the principle of specificity which states that exercise of a specific type, intensity and duration that utilizes a specific muscle group will elicit distinct physiological adaptions which in turn highlights the importance of sport-specific testing [18]. When using lower limb dominant equipment such as a treadmill or a cycle ergometer, the use of the larger leg muscles will demand greater oxygen consumption than the smaller upper limb muscles therefore resulting in a greater VO_2peak_ [3,8,18,32]. From this previous research, it can be deduced that even within the same person peak oxygen uptake is specific to a given type of activity. Therefore, to obtain relevant values, emphasis should be placed on the testing methods and its specificity to the activity of the participant. Consequently, when assessing the VO_2peak_ of surfers the SWB should be the standard and preferred testing method.

Peak aerobic power outputs in this study were found to be less than previous studies. The mean peak aerobic power output (W) in this study was 69.91 (±19.33) W which was less than the 101.26 (±18.49) W and 199 (±24) W reported by Furness, et al. [6] and Loveless and Minahan [10], respectively. The authors hypothesize the discrepancies in peak aerobic power outputs may be attributed to the differences in computation algorithms used by the SwimFast and Vasa ergometers however this was outside the scope of this study.

Taking into account the demanding activity of arm paddling in surfing, Meir, et al. [5] reported that during one hour of recreational surfing, the mean HRs during arm paddling represented 80% of the laboratory HR_peak_ attained by the surfers during a progressive SWB ergometer VO_2peak_ test. The mean laboratory SWB HR_peak_ in this study was found to be 164.78 (±13.55) b·min^−1^ with an associated mean percent of age-predicted HR_peak_ of 88.11% (±7.79) (Table 2). Meir, et al. [5] reported a mean HR_peak_ of 180 (±6) b·min^−1^ and mean percent of age-predicted HR_peak_ of approximately 90.5%. The results by Meir, et al. [5] compared favorably to Loveless and Minahan [10] who reported a mean HR_peak_ of 194 (±5) b·min^−1^ and mean percent of age-predicted HR_peak_ of approximately 96%. Furness, et al. [6] reported a mean peak HR of 175.58 (±10.51) b·min^−1^ with an approximate percent of age-predicted HR_peak_ of 90.74%. The similarities in values of mean percent of age-predicted HR_peak_ between the present study and previous research points to similarities between the protocols used in terms of eliciting similar cardiovascular demands on the participants.

### 4.3. Multiple Regression Model

The final aim of this study was to propose a regression model to predict SWB ergometer VO_2peak_ values. This regression model would serve to provide recreational surfers with no access to an SWB ergometer the opportunity to predict their surfing specific VO_2peak_.

After satisfying the eight assumptions of a multiple regression analysis, it was determined that the three independent variables (predictors) best included in the model were: (1) treadmill VO_2peak_ (mL/kg/min); (2) surf experience (years); and (3) hours surfed (per week). The models R^2^ value of 0.863 infers that these three independent variables can explain 86.3% of the variability in the dependent variable (SWB ergometer VO_2peak_). The model’s adjusted R^2^ value of 0.726 corrects for positive bias to provide a value that would be expected in the population. Therefore, when generalized to a larger population, the independent variables in this model explain 72.6% of the variability in the dependent variable. Given the development of this equation, further research should aim to conduct profiling on a larger recreational cohort to further assess its validity.

## 5. Study Limitations and Strengths

This pilot study is the first to explore the novel idea of producing a regression model to predict surf specific SWB VO_2peak_ values in a recreational surfing cohort. This study acts as a requisite initial step in exploring the use of such a regression model in a larger recreational surfing cohort and paves the path for further study into producing a similar model for a larger competitive surfing cohort. In addition to being the first pilot study to produce a regression model, this study is one of few to profile recreational surfers on both a SWB ergometer and a treadmill.

The authors acknowledge the regression model produced is not without limitations and further study is warranted in the area. It is important to note that the model is only relevant to the current sample and therefore caution should be exercised when using this model in age groups and surfing populations outside of the current study. Further research on a significantly larger sample size is necessary to validate the regression model to allow generalization and application outside of this study.

## 6. Conclusions

The present study is the first to use the SwimFast ergometer in VO_2peak_ testing in recreational surfers and compare its values to those from the gold-standard treadmill within the same sample. The authors propose that the results will help in bolstering the current knowledge of available tools for physiological assessments in the surfing population. Furthermore, the present study was the first to produce a regression model for predicting SWB ergometer VO_2peak_. The regression model which uses surfing experience (years), hours surfed (per week) and treadmill VO_2peak_ values (mL/kg/min) could allow surfers with no access to an SWB ergometer to predict their surfing specific VO_2peak_. This could allow the surfer to compare their peak aerobic fitness with other recreational and elite surfers allowing them to identify strengths or deficiencies in their aerobic power and potential goals for improvement.

## Figures and Tables

**Table 1:** Physical Attributes and Experience of Recreational Surfers M ± SD (*n* = 9).

Measure	Value
Height (cm)	176.90 ± 3.97
Mass (kg)	78.37 ± 10.16
BMI (kg/m^2^)	24.98 ± 2.28
Surfing Experience (years)	12.89 ± 7.82
Surfing Frequency (hours per week)	5.22 ± 2.43

**Table 2:** Key Performance Variables for Recreational Surfers M (±SD).

Measure	Treadmill	Swim Bench	*p* Value
VO_2peak_ (L/min)	5.16 (±0.86)	2.98 (±0.89)	<0.001
VO_2peak_ (mL/kg/min)	66.01 (±8.23)	37.41 (±8.73)	<0.001
VCO_2_	5.69 (±1.12)	3.48 (±0.89)	<0.001
VE	134.20 (±25.60)	92.15 (±17.61)	<0.001
RQ	1.12 (±0.068)	1.28 (±0.10)	<0.001
HR_peak_ (b·min^−1^)	184 (±10)	165 (±14)	0.004
Percent of age predicted HR_peak_ (%)	98.53 (±4.61)	88.11 (±7.79)	0.003
Peak aerobic power (W)	N/A	69.91 (±19.33)	N/A

**Table 3:** Summary of Multiple Regression Analysis.

Variable	B	SE_B_	β
Intercept	−33.027	18.007	
Surf experience (years)	0.480	0.260	0.428
Hours surfed (per week)	3.968	1.616	0.547
Treadmill VO_2peak_ (mL/kg/min)	0.699	0.239	0.676

B = unstandardized regression coefficient; SE_B_ = standard error of the coefficient; β = standardized coefficient.

## Appendix A

Literature review and appraisal were conducted using the search terms “Surf*”, “Cardiopulmonary Fitness”, “VO_2max_”, “Peak VO_2_”, “Ergometer” and “Treadmill”. These search terms were combined through the use of Boolean operators “AND” and “OR” to limit and refine the search. This search provided an awareness of the existing literature on the topic and was used to inform the formal search criteria.

A two-tiered search strategy was then implemented. First, a comprehensive search of online databases including PubMed, CINHAL, SPORTSDiscus, and EBSCO was completed. An identical search string was used in each database, and no limits were applied. Search results were compiled in Endnote, and duplicates removed. Second, reference lists of key articles and reviews identified by the search were pearled in order to identify additional relevant papers. All articles were screened for inclusion using the following defined criteria: (1) published in English; (2) involved human participants; (3) participants mean age >18 years; (4) participants >12 months experience in surfing; (5) the article specifically investigated VO_2peak_ using an ergometer or treadmill (6) oxygen consumption was analyzed using a gas analysis system meeting the Australian Institute of Sport accreditation standards for precision and accuracy; (7) the article contained original data. For this study, VO_2peak_ was defined as the highest rate at which oxygen could be taken up and consumed by the body during intense exercise. Ergometer was defined as an apparatus that measures work and energy expenditure during a period of physical exercise.

The methodological quality of the articles selected for inclusion was assessed using the Joanna Briggs Institute (JBI) Appraisal Checklist for Cohort Studies. The JBI Appraisal Checklist employs an 11-question checklist to determine the extent to which the study has addressed the possibility of bias in its design, conduct and analysis [34]. The checklist is comprised of closed answer questions where a “yes” is awarded 1 point and a “no” or “unclear” is awarded 0 points. A modified version of this tool was used where questions 2, 7, 8, 9 and 10 were removed, as they were designed to evaluate aspects of methodological quality that did not align with the design of the included articles. All studies were independently rated by the authors with the level of agreement measured using a Cohen’s Kappa analysis of all raw scores (6 scores per paper). For final scores, any disagreements in points awarded were settled by consensus. Due to the modifications made to the JBI checklist, all scores were first converted to percentages to enable grading as proposed by Lyons, et al. [35]. On this basis, the grading criteria applied when rating the methodological quality of the included studies were as follows: JBI total score <61%, ‘poor’ methodological quality; >61%, ‘good’ methodological quality. Following the critical appraisal of the included studies, key data were extracted and tabulated from all included studies.

Search String for PubMed, CINHAL, SPORTSDiscus, and EBSCO: (((cardiopulmonary response OR cardiovascular response OR oxygen consumption OR physiological impact OR physiological capacity OR physiological response OR physiological effects OR physiological profile OR maximal aerobic power OR aerobic OR performance OR physiological Parameter* OR peak power output)) AND (treadmill OR maximal exercise testing OR graded exercise testing OR high intensity testing OR VO_2max_ test OR VO_2max_ OR VO_2peak_ test OR VO_2peak_ OR swim bench OR ergometer OR ergometers OR ergometric)) AND (Surf*).
